# A Nanoparticle-Based Approach for the Detection of Extracellular Vesicles

**DOI:** 10.1038/s41598-019-46395-2

**Published:** 2019-07-11

**Authors:** Md. Khirul Islam, Parvez Syed, Laura Lehtinen, Janne Leivo, Kamlesh Gidwani, Saara Wittfooth, Kim Pettersson, Urpo Lamminmäki

**Affiliations:** 1 Department of Biochemistry, Division of Biotechnology, University of Turku, Turku, Finland; 2 Department of Pathology, University of Turku and Turku University Hospital, Turku, Finland; 3 Department of Urology, Erasmus Medical Center, Rotterdam, The Netherlands

**Keywords:** Prostate cancer, Diagnostic markers

## Abstract

The analysis of extracellular vesicles (EVs) typically requires tedious and time-consuming isolation process from bio-fluids. We developed a nanoparticle-based time resolved fluorescence immunoassay (NP-TRFIA) that uses biotinylated antibodies against the proteins of tetraspanin family and tumor-associated antigens for capturing EVs from urine samples and cell culture supernatants without the need for isolation. The captured-EVs were detected either with Eu^3+^-chelate or Eu^3+^-doped nanoparticle-based labels conjugated either to antibodies against the tetraspanins or lectins targeting the glycan moieties on EVs surface. The NP-TRFIA demonstrated specific capturing and detection of EVs by antibodies and lectins. Lectin-nanoparticle based assays showed 2–10 fold higher signal-to-background ratio compared with lectin-chelate assays. The nanoparticle assay concept allowed surface glycosylation profiling of the urine derived-EVs with lectins. It was also applied to establish an assay showing differential expression of tumor-associated proteins on more aggressive (higher ITGA3 on DU145- and PC3-EVs) compared to less aggressive (higher EpCAM on LNCaP-EVs) PCa- cell lines derived-EVs. This NP-TRFIA can be used as a simple tool for analysis and characterization of EVs in urine and cell culture supernatants. Such approach could be useful in identification of disease-specific markers on the surface of patient-derived urinary EVs.

## Introduction

Extracellular vesicles (EVs) are 40 to 1000 nm sized membranous particles which are secreted by most of the cells and found in the bodily fluids like urine, plasma and saliva^1^. The secreted EVs contain transmembrane and cytosolic proteins and nucleic acids. EVs play a crucial role in cell-to-cell communication and promote cancer progression and metastasis through the cargo they carry^2,3^. EVs may reflect the pathophysiological condition of the cells they originate from and thus may hold a key for a breakthrough in the field of non-invasive diagnostics^4^.

Urinary EVs are considered as potential biomarkers for certain urogenital tract-associated diseases such as prostate cancer. Traditionally, EVs are isolated for analysis by differential ultracentrifugation of the urine sample. However, this process is labor-intensive and requires large volumes of urine as well as an expensive laboratory set-up^5^. Other common methods used for the isolation of EVs include filtration using nanomembranes, precipitation using aggregating agents and size-exclusion chromatography (SEC), which have their own sets of limitations including co-isolation of unwanted proteins and large volumes of sample needed^6,7^.

The surface of the EVs is enriched with integral membrane proteins of tetraspanin family, such as CD9, CD63 and CD81^8^ and also with mannose- and sialic acid-containing glycoproteins^9^. In an evaluation study made by Duijvesz *et al*. tetraspanins were used as targets for antibody binding to analyze EVs in various biofluids^10^. Besides tetraspanins, other glycoproteins on EVs can be used to detect and characterize different EV subpopulations. Altered glycosylation of glycoproteins is a common phenomenon seen in many diseases, like cancer^11^, classical galactosaemia^12^, and polycystic kidney disease^13^. Lectins, glycan binding proteins, can be used as tools for the detection of various carbohydrate structures including the cancer associated altered glycosylations^14^. Indeed, lectin microarrays have been used in various studies for the surface glycosylation profiling of the EVs of urine^13,15^. However, such approach requires purified EVs from large sample volumes to work with.

The aim of our study was to develop a simple assay format which enables the detection and characterization of EVs by the protein and glycan content on their surface without the need for extensive purification steps. To achieve this, we explored the use of various reporter molecules targeting EVs, which include antibodies specific to tetraspanin family proteins and tumor-associated membrane antigens as well as lectins recognizing the glycan moieties. We used solid surface immobilized antibodies for capturing EVs and compared the performance of two types of lanthanide based tracers i.e., Eu^3+^-chelates and Eu^3+^-doped nanoparticles conjugated to antibodies or lectins. The assays were tested for surface glycosylation profiling of urinary EVs and for the characterization of EVs derived from prostate cancer cell line medium.

## Results

### Evaluation of NP and Eu^3+^-chelate based tracers

**Figure 1:**
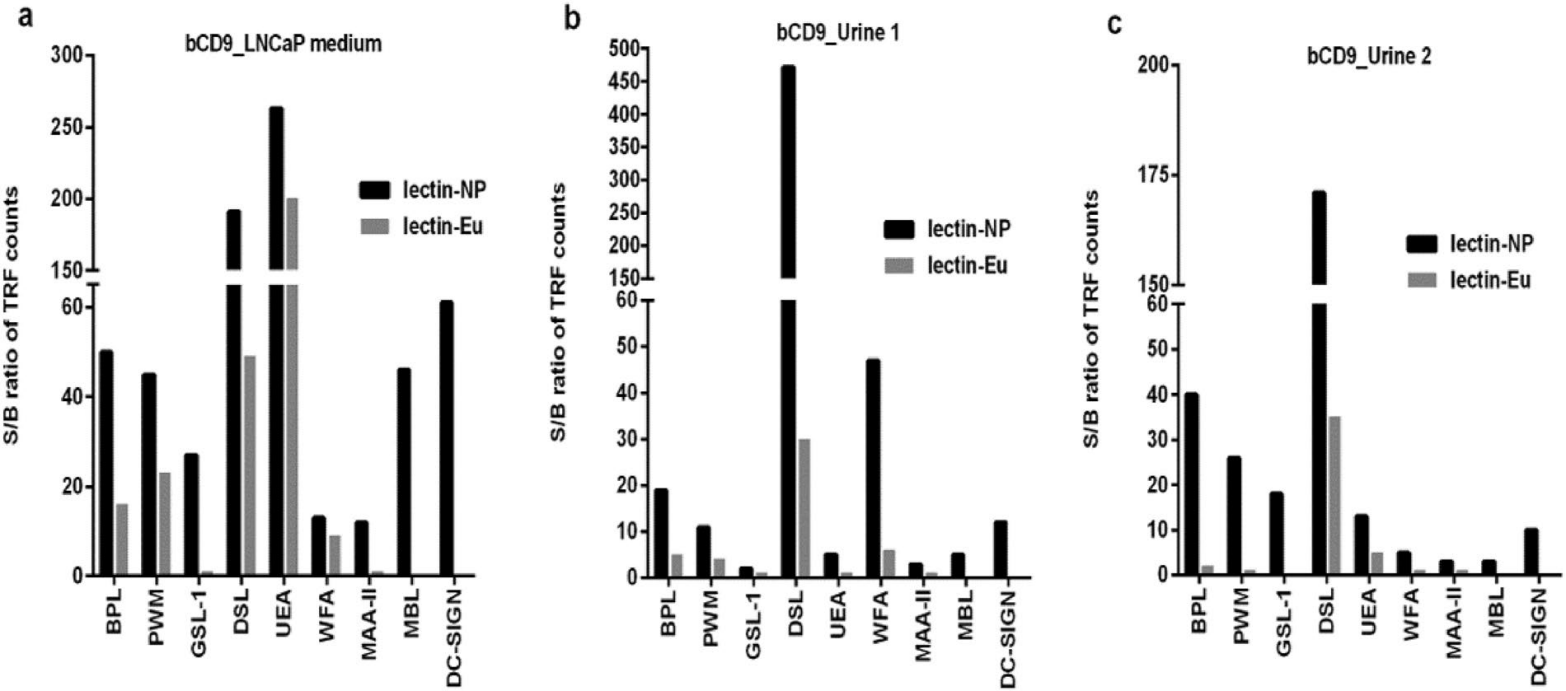
Performance of Eu^3+^-nanoparticles (Eu-NP) and Eu^3+^-chelate as tracers (labels). EVs were captured from LNCaP supernatant and individual urine samples with biotinylated anti-CD9 mAb immobilized onto streptavidin-coated microtiter wells and detected with either Eu-NP or Eu-chelate based tracers. (**a)** Comparison of S/B ratios obtained from lectin-NP and lectin-Eu assays in LNCaP supernatant. (**b,c)** Comparison of S/B ratios from lectin-NP and lectin-Eu assays in two healthy urine samples, respectively. All assays were performed in triplicate and average value was taken for analysis. For the lectins MBL and DC-SIGN, only NP tracer was used.

Different time-resolved fluorescence (TRF) based immunoassay configurations were set up in order to explore the performance of the tracers for the detection of EVs. An immobilized anti-CD9 antibody was used for capturing EVs in all these assays, whereas tracer varied being either Eu^3+^-nanoparticles or Eu^3+^-chelate conjugated to anti-CD9 antibodies (antibody-NP and antibody-Eu assays) or lectins (lectin-NP and lectin-Eu assays). These assays were applied to the detection of EVs from the cell culture supernatant of LNCaP cells and urine samples of two healthy volunteers. Fresh cell culture medium and EV-stripped urine served as background samples. The signal-to-background (S/B) ratios obtained from the assays were compared. Antibody-NP and antibody-Eu tracers showed similar performance with all the sample materials tested (Supplementary Fig. S1). The S/B ratios obtained from lectin-NP tracers were systematically 2–10 folds higher compared to lectin-Eu tracers (Fig. 1a–c), even though the S/B ratios obtained with NP-tracers varied considerably depending on the lectin used. Some of the lectins like GSL-1 and MAA-II detected the EVs only when conjugated with NPs but not with Eu^3+^-chelates.

### Specificity of nanoparticle aided TRFIA

Next, we explored the performance of the nanoparticle aided TRFIA (NP-TRFIA) where microtiter wells immobilized with anti-CD9 antibody were used for capturing EVs from LNCaP cell culture supernatant, and as a tracer to detect the captured EVs, NPs conjugated either with tetraspanins antibodies (CD9-, CD81-, and CD63-NP) or lectins (MBL-NP and DC-SIGN-NP) were used. To assess the specificity of binding NPs conjugated with antibodies against proteins which are not usually present on the surface of EVs (mAb 5E4, specific for Prostate specific antigen PSA and kallikrein 2) were included in the assay. The unspecific binding in the assay was also evaluated in the absence of any capture or tracer antibody, and in the presence of capture antibodies against another molecule not present on the surface of EVs (mAb 10B5, specific for mycotoxin).

**Figure 2:**
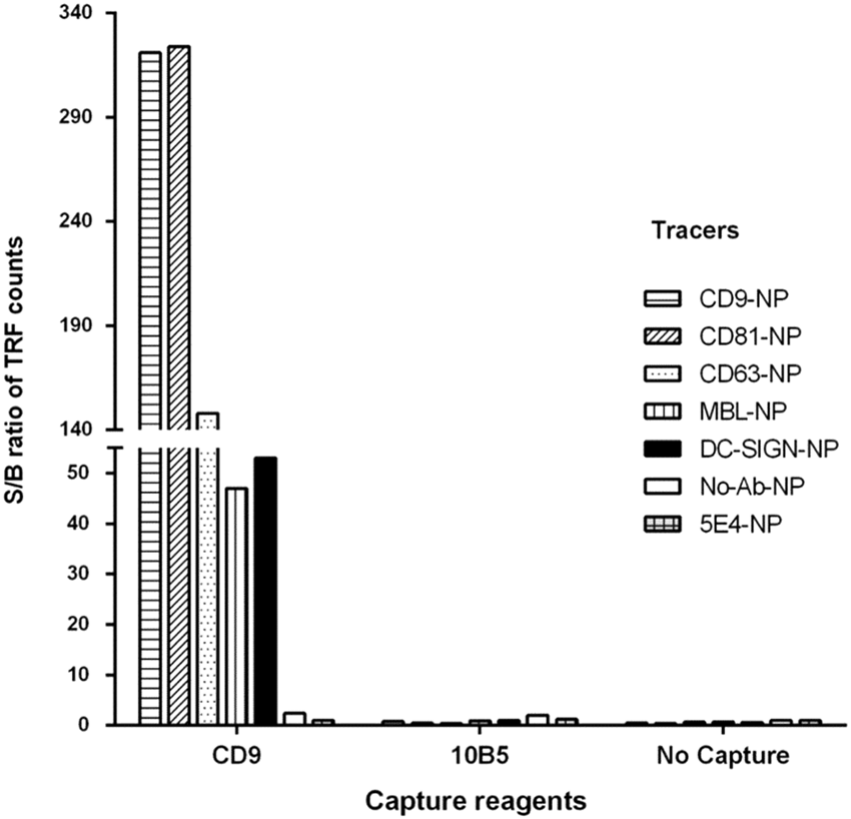
Capture and tracer specificity of nanoparticle based TRFIA (NP-TRFIA). Several different tracers (CD9-, CD81-, CD63-, MBL-, and DC-SIGN-NP) were evaluated for their capacity to detect EVs captured from LNCaP supernatant by immobilized anti-CD9 antibody. The immobilized non-EVs specific antibody 10B5 (anti-Deoxynivalenol) and a reaction without any capture antibody (No capture) were used to rule out non-specific binding. Additional controls included tracer NPs without any antibody or lectin (No-Ab-NP) and NPs coated with another non-EVs specific antibody 5E4 (5E4-NP).

Significant signals were observed only from the assays where the immobilized anti-CD9 antibody was used for capturing the EVs followed by detection with either one of the anti-tetraspanin antibody or lectin coated–nanoparticle tracers. The signals obtained with non-specific antibodies (5E4 or 10B5) did no differ significantly from those obtained from the control without any capture (No Capture) and tracer (No-Ab-NP) (Fig. 2).

### Sensitivity and linearity of NP-TRFIA

**Figure 3:**
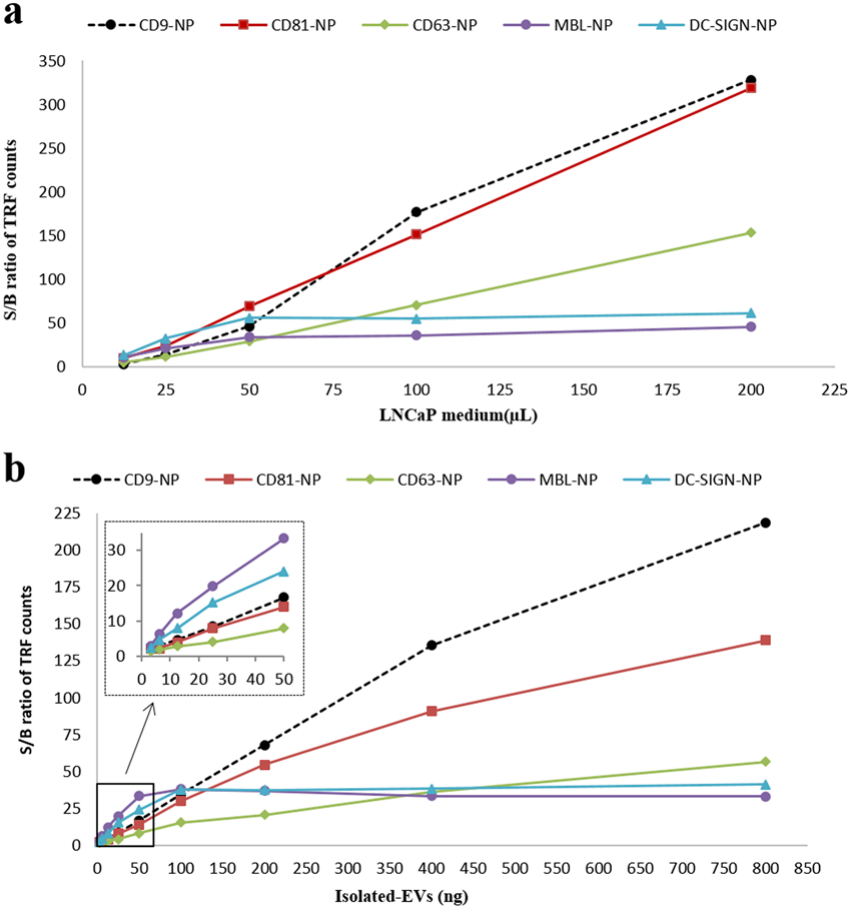
Sensitivity and linearity response of the assay based on NP tracers. (**a**) LNCaP medium dilution series and (**b**) isolated EVs from LNCaP medium analyzed with an assay using biotinylated anti-CD9 for EVs capture and either with anti-CD9-, anti-CD81-, anti-CD63-, MBL- or DC-SIGN-NP as a tracer.

To evaluate the sensitivity and linearity of the NP-TRFIA assay, we performed an experiment with serial dilutions of both LNCaP cell culture supernatant and EVs-isolated from LNCaP supernatant. The dilutions were made in RPMI medium. The response curves in Fig. 3a,b show the increase of S/B ratio along with the increasing amount of LNCaP cell culture medium or isolated EVs, respectively, in the well. The amount of isolated LNCaP-EVs which could be detected using DC-SIGN-NP and MBL-NP was found to be 1–2 ng/mL. Detection limit of isolated LNCaP-EVs using CD9-NP and CD81-NP was 4–5 ng/mL. However, for the detection of LNCaP-EVs using CD63-NP, more than 13 ng/mL of isolated EVs were needed (Fig. 3b). The reproducibility of the assay was analyzed where the anti-CD9 capture and different NP tracers were tested in two different days, for both LNCaP and urine media. Very similar specific signal levels were obtained in each assay at the both times (Supplementary Fig. S4).

### Glycoprofiling and tetraspanin characterization of urine EVs

Various combinations of biotinylated capture antibodies and NP-tracers were used to characterize EVs from urine. We used all the possible combinations of three anti-tetraspanin (anti-CD9, -CD81 and -CD63) capture antibodies and corresponding NP tracers as well as a panel of lectin-NP tracers (Table 1) to detect uEVs from minimally preprocessed (15 min at 4000 g) 12 individual urine samples (six female and six male samples). EVs-stripped urine from a pool of 12 urine samples was used as a blank.

**Table 1:** List of lectins used in this study and their carbohydrate binding specificities.

Lectin name	Abbreviation/synonym	Major carbohydrate binding specificity
*Bauhinia purpurea* lectin^a)^	BPL/BPA	N-acetylgalactosamine, lactose
*Datura Stramonium* lectin^a)^	DSL	(β-1,4) linked N-acetylglucosamine oligomers
*Griffonia (Bandeiraea) simplicifolia-*1 lectin^a)^	GSL-1/BSL-1	N-acetylgalactosamine, galactose
*Maackia amurensis* agglutinin II^a)^	MAA-II/ MAH	α2-3-linked sialic acids
*Phytolacca Americana* (pokeweed) lectin^a)^	PWM/Pokeweed mitogen	di-N-acetylchitobiose
*Ulex europaeus* agglutinin^a)^	UEA-1	Fucα1-2Gal
*Wisteria floribunda* agglutinin^a)^	WFA/WFL	GalNAcα or β- 3 or 6 position of galactose
Dendritic Cell-Specific Intercellular adhesion molecule-3-Grabbing Non-integrin^b)^	DC-SIGN	Nonsialylated Lewis antigens and high mannose-type structures
Mannose-binding lectin^b)^	MBL	Mannose

^a)^Lectins conjugated with Eu^3+^-chelates and NPs. ^b)^Lectins conjugated with NPs only.

**Figure 4:**
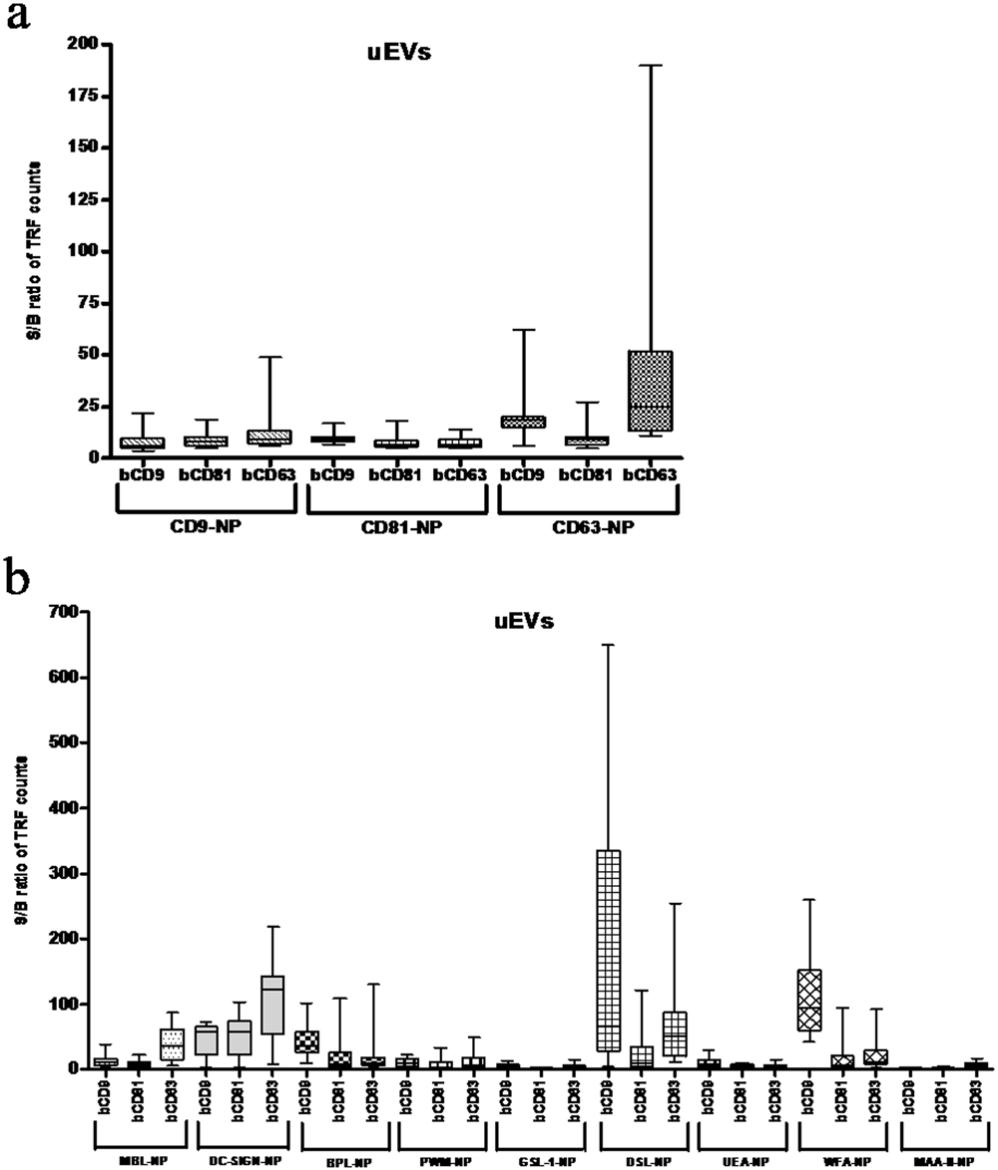
Profiling of urine EVs (uEVs) using NP-TRFIA with antibody-NPs and lectins-NPs. EVs from healthy urine samples (male *n* = 6, female *n* = 6) were first captured with biotinylated antibodies (bCD9, bCD81 or bCD63) and then detected with NP tracers. (**a)** Represents the assays with CD9-, CD81-and CD63-NP and (**b)** the assays with lectin-NP tracers.

The S/B ratios obtained from the assays involving CD9-NP and CD81-NP showed minor variation upon using either biotinylated anti-CD9, -CD81 or -CD63 antibodies for capturing the urinary EVs (uEVs) from the samples while CD63-NP assay had higher S/B ratios variation among the individual urine samples (Fig. 4a). Signal intensities obtained from different lectin-NPs showed distinct variation from each other. Highest S/B ratios were seen with assays using lectins DC-SIGN-NP (in bCD63-DC-SIGN combination), DSL-NP (in bCD9-DSL combination), and WFA-NP (in bCD9-WFA combination) (Fig. 4b). Semiquantitative comparison of the assay results on basis of the sex of the sample donor showed that levels of EV associated tetraspanins (CD9, CD81, CD63) were consistently higher among the male samples (Supplement Fig. S3).

### Capturing through tumor-associated proteins on the EVs-surface

The NP-TRFIA assay was evaluated for its efficacy in the identification of known tumor-associated proteins (EpCAM and ITGA3) on the surface of EVs. Biotinylated anti-EpCAM, anti-ITGA3 or anti-CD63 antibodies were applied for capturing EVs from cell culture supernatants of the prostate cancer cell lines LNCaP, DU145 and PC3 and one of the control cell line HEK293. The captured EVs were detected using CD63-NPs.

**Figure 5:**
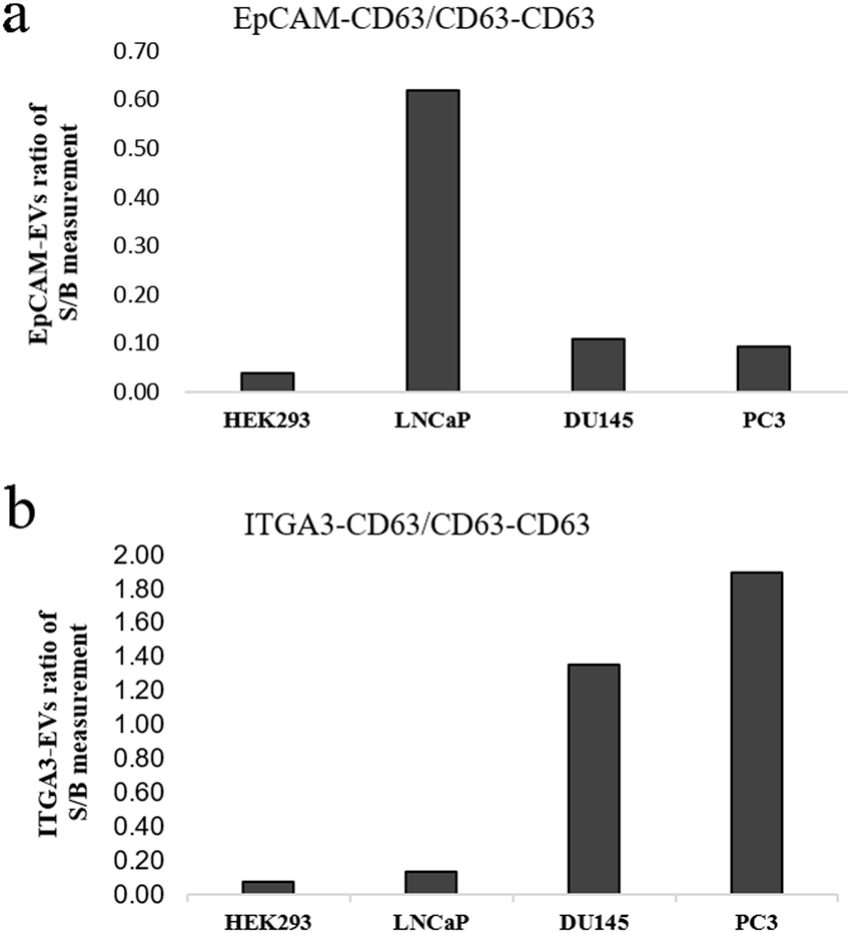
Tumor-associated proteins on the surface of EVs. Expression of tumor-associated proteins EpCAM and ITGA3 were studied on the EVs in cell culture supernatants of less aggressive prostate cancer cell line LNCaP and more aggressive prostate cancer cell lines DU145 and PC3. Figures (**a,b)** represent the assays where bEpCAM and bITGA3 antibodies were used for capturing the EVs, respectively. In all cases, CD63-NP was used for the detection of the captured EVs. Here, S/B obtained from EpCAM-CD63 and ITGA3-CD63 were normalized by the S/B ratio from CD63-CD63.

In the CD63-CD63 assay, the expression of CD63 was relatively similar among the EVs of the prostate cancer cell lines (data not shown). For relative quantification of EpCAM-and ITGA3-EVs in the experiment, the S/B ratio obtained from EpCAM and ITGA3 assay was normalized with the CD63-CD63 results (total EV). After normalization, 6–7 fold higher expression of EpCAM was seen with the EVs of less aggressive prostate cancer cell line LNCaP compared to more aggressive DU145 and PC3 cell lines (Fig. 5a). On the other hand, the EVs of androgen-independent and more aggressive DU145 and PC3 cell lines showed 10–15 fold higher expression of ITGA3 compared to androgen-dependent LNCaP cell line (Fig. 5b).

## Discussion

EVs have gained considerable amount of attention in the past decade, with large number of studies ranging from basic cell biology to translational applications. The characterization of the EVs often require labor intensive isolation steps, or commercial kits for downstream analysis. In this study, we reported a lanthanide chelate-doped nanoparticle aided TRFIA (NP-TRFIA) approach to detect the presence of specific proteins and glycans on the surface of EVs from minimally preprocessed urine samples and cell culture supernatants. The EVs were first specifically captured using solid surface-immobilized antibodies against tetraspanins or tumor-associated proteins. The captured EVs were then targeted for the tetraspanins or the glycan moieties present on their surface using anti-tetraspanin antibodies or lectins coated on the nanoparticles, respectively, for time-resolved fluorescence based detection. The salient features of the NP-TRFIA are: (1) the approach is robust, simple and sensitive for the detection of EVs directly from urine. (2) the EVs can be detected through the glycan moieties presented on their surface using lectins as binders. (3) For lectin binders, NP labels provide a clear advantage over the Eu-chelates based labels in the detection of glycans on EVs (4) the assay can identify the tumor-associated proteins on the surface of EVs.

Currently, to identify specific surface marker proteins of EVs, immunoblotting and flow cytometry analysis are considered as a gold standards. Both flow cytometry and western blotting always demand isolated EVs for the analysis, and, unfortunately, the standard method for isolation of EVs is time consuming, instrument-dependent and labor-intensive^16^. In addition, immunoblotting is a quite laborious method. On other hand, major limitation of flow cytometry for analyzing EVs is that the EVs are too small to fit in the detection range of most of the flow cytometer instruments^17^. Thus, antibody coated magnetic or latex beads are used to capture and carry the vesicles into the flow cytometry and to get them visualized by laser^18^. Using the NP-TRFIA approach, we were able to capture and detect EVs directly from minimally preprocessed urine sample involving a simple centrifugation step. Since the EVs, which can carry molecular information on the tissue of their origin, are basically released from all the cells of urogenital tract into urine, this simple method for urine EVs testing may find applications in the development of non-invasive diagnosis for diseases associated with urogenital tract tissues^19^.

In a previous study, Duijvesz *et al*. showed that the use of the same monoclonal tetraspanin specific antibody for the capture and detection, it is possible to quantify EVs from various biomaterials without any preanalytical processing^10^. We aimed to expand the previously described TRFIA both in terms of the assay sensitivity and specificity. First, to increase the sensitivity of the TRFIA, we used high-performance Eu^3+^-doped nanoparticles as reporters. Embedding ca. 30000 Eu^3+^-chelates for time-resolved fluorometry based detection, the nanoparticles labels provide extremely high specific signaling activity offering potential for improved detection sensitivity as compared to molecular labels based bioaffinity assay techniques^14,20,21^. For example, Logozzi *et al*. developed an ELISA based assay that could detect purified exosomes from plasma up to 3–10 µg (60–200 ng/mL)^22^ whereas our nanoparticle-based TRFIA could detect isolated EVs up to 1–13 ng (0.03–0.06 ng/mL) (Fig. 3). Secondly, to broaden the specificity of the assay, we utilized a selection of lectins with various glycan specificities to detect glycosylations on the surface of the EVs. Furthermore, the use of lectin-NPs as reporters has previously been shown to increase the cancer specificity of conventional immunoassays, especially in the case of highly glycosylated proteins, such as CA-125 (*i.e*. mucin-16)^14^. Since the lectin–glycan interactions are usually weak (dissociation constant, K_d_ = 10^−4^–10^−7^ M) in comparison to the antigen–antibody interactions (K_d_ = 10^−8^–10^−12^ M), the multivalency gained from the nanoparticle coating has proven to be crucial for some lectins^23^.

Altered protein glycosylation is a common phenomenon in cancer. Detection of altered glycans potentially present on the surface of EVs could be a mean for the identification of tumor cell derived EVs for diagnostic purposes. Previously, the surface glycosylation profiling of the uEVs have been explored using lectin microarrays which requires purification but also labeling of uEVs^13,15^. Kosanović and Janković^24^ also used immobilized purified EVs from urine and probed them with various biotinylated plant lectins using ELISA based detection with a streptavidin-horseradish peroxidase conjugate. By circumventing the tedious process of isolation and labeling of the EVs, NP-TRFIA provides a straightforward approach to explore cancer-associated glycosylation patterns on the EVs in urine and possibly in other biofluids. A common hindrance in the evaluation of uEVs is the presence of THP in the urine. In order to deplete THP, we treated urine samples with NaCl, which resulted in partial removal of THP. The S/B ratios obtained with NP-tracer based assays were very similar in the NaCl treated and untreated urine samples (Supplementary Fig. S2), thus indicating the presence of THP had not impact on the outcome of the assay.

We also evaluated the efficacy of the developed NP-TRFIA assay to determine the abundance of tumor-associated proteins on EVs. It has been reported that the overexpression of EpCAM is associated with the progression of prostate cancer^25^. In a previous study by Xu *et al*.^26^, it was shown that the EpCAM overexpression correlates with the hormone (androgen)-dependency of prostate cancer. Here, we showed that EpCAM is overexpressed on the EVs of hormone-dependent LNCaP compared to those of hormone-independent DU145 and PC3 cell lines. Similarly, Bijnsdorp *et al*. reported an increased level of ITGA3 on the surface of urine EVs in metastatic prostate cancer using Western blot and a small cohort of samples from three prostate cancer patients^27^. Through our simple NPs-based assay, we reaffirmed their findings by showing higher expression of ITGA3 on the EVs obtained from more aggressive prostate cancer cell lines DU145 and PC3 compared to the less aggressive prostate cancer cell line LNCaP and HEK293 control. Our results suggest that our assay concept could be used for the evaluation of other tumor-associated surface proteins as well and highlight the potential of this assay for diagnostic and disease monitoring applications.

There is great potential for using EVs as biomarkers for cancers and other diseases. However, this requires highly sensitive and cost-efficient detection methods. Our novel NP-TRFIA enhances the possibilities to detect EVs with required sensitivity from minimally processed urine samples. It creates an open platform for testing different combinations of capture and detection reagents, including antibodies and lectins, against tumor-associated surface epitopes for biomarker discovery. With the right combination of capture and tracer, this assay has the potential for liquid biopsies via EVs, thereby possibly improving the diagnosis/prognosis of different diseases.

## Materials and Methods

### Cell culture supernatant

We used EVs derived from the cell culture supernatant from LNCaP, DU145 and PC3 prostate cancer cell lines. These cell lines were cultured in T175 culture flasks (Greiner Bio-One, Fricken-hause, Germany) in RPMI 1640 (Lonza, Verviers, Belgium) supplemented with 10% fetal bovine serum (FBS) (GIBCO, USA) and 1% penicillin/streptomycin (P/S). Upon reaching the confluency of 80–100%, supernatant was collected.

### Isolation of EVs from LNCaP cell culture supernatant

For the enrichment of EVs, a protocol described by Welton *et al*. was used with slight modifications^28^. In brief, the cell culture supernatant was subjected to centrifugation at 4000 × g at +4 °C for 15 min, followed by passing through a 0.22 µm filter to remove cell debris. The supernatant was then ultra-centrifuged at 200,000 × g for 2 h at +4 °C (QuickSeal tube, 90 Ti Fixed angle rotor, Beckman coulter). The supernatant was discarded and the pellet was resuspended in 100 µL of PBS. The total protein concentration of the resuspended pellet was determined using NanoDrop^TM^ (measuring absorbance at 280 nm, in duplicates). The isolated EVs were used as a standard preparation for determining assay sensitivities.

### Ethical approval

Ethical permission for this study was obtained from the University of Turku ethics committee (statement number 56/2018). Voluntary donation of healthy urine samples was collected with written consent from the lab members of biotechnology division, University of Turku. Informed consent was obtained from all individual participants involved in the study. All methods were performed in accordance with relevant guidelines and regulations.

### Urine samples

First morning mid-stream urine samples from twelve healthy volunteers were collected: 6 male (median: 31 years; standard deviation (SD) ± 4.4 years) and 6 female (median: 29 years; SD ± 5.4 years). Preprocessing of the urine sample was done by storing the urine samples at +4 °C for one day and then clearing the cell debris from the samples by centrifuging the samples at 4,000 × g for 15 min at +4 °C. The cell-free urine samples were stored at −80 °C until use.

### EVs-stripped urine

EVs-stripped urine was produced by passing a pool of urine samples through the ultrafiltration concentrator Vivaspin 2 column (polyethersulfone nanomembrane, MWCO 100 kDa, Sartorius, Germany) was used as blank when performing the assays using urine samples. This was done by centrifuging the sample containing column at 2,000 × g for 10 min. Thus collected urine flow through should not contain any EVs since 100 KDa cut-off filter does not pass any particles larger than 10 nm and EVs lie in the range from 40–1000 nm. The column was washed with 1X PBS prior to use to remove glycerol and preservatives from the column.

### Depletion of Tamm-Horsfall protein (THP) from urine

The precipitation of THP from individual urine sample was done using the protocols described by Kosanovic *et al*.^24^ and Zhou *et al*.^29^, with slight modifications as follows. Briefly, to the cell-free urine sample, 0.58 M NaCl was added and incubated for 2 h at RT. The sample was then centrifuged at 3,000 × g at RT for 25 min. The supernatant was carefully collected and used for immunoassays.

### Sensitivity determination of assay

The limit of detection of NP-TRFIA assay was determined based on signal-to-background approach. The assay sensitivity was determined by multiplying standard deviation (SD) of blank with three (3) and then the value was divided by the slope of the standard curve at the linear response range.

### Biotinylation of capture antibodies

Monoclonal antibodies (mAbs) anti-CD9 (clone 209306), anti-EpCAM (clone 158210), and anti-integrin alpha 3 (anti-ITGA3, clone IA3) were purchased from R&D systems (Abingdon, UK). Anti-CD81 (clone 555675) and anti-CD63 (clone 556019) were purchased from BD Bioscience (Vantaa, Finland). 10B5 (the division of Biotechnology, University of Turku), an antibody against mycotoxin, was used as a control. These mAbs were biotinylated as previously described with slight modifications^30^. Briefly, biotin isothiocyanate (BITC) was dissolved in dimetyl formamide (DMF) to a final concentration of 10 mM. The pH of the antibody solution was adjusted to 9.8 with 50 mM of carbonate buffer. To the final reaction volume (final mAb concentration: ~2 mg/ml), 40-fold excess of biotin over mAb was added and the reaction was incubated for 4 h at RT. Unreacted BITC was removed by gel filtration with a NAP-5, and NAP-10 columns (GE-Illustra, Diegem, Belgium) using 50 mmol/L Tris-HCL (pH 7.5), containing 150 mmol/L NaCl and 0.5 g/L NaN_3_ and then biotinylated antibodies were stored with 1 g/L BSA at +4 °C.

### Preparation of nanoparticle-bioconjugates

For the preparation of NP-bioconjugates, NPs (Seradyn Indianapolis IN, USA) which are monodispersed and carboxyl-modified fluoro-max polystyrene beads were used. These NPs yield a long-lifetime fluorescence equivalent to ~30,000 Eu^3+^ per particle^20^. Anti-CD9, -CD81 and -CD63-antibodies and lectins (Table 1) were covalently coupled to activated carboxyl groups present on the NPs^14,21^. The mAb 5E4 (the division of Biotechnology, University of Turku), an antibody specific for PSA and human kallikrein 2, was also conjugated with NPs and used as a control. The concentration of antibodies and lectins in the coupling reaction was 0.1 mg/mL. The NPs (10^12^) were washed with 10 mmol/L phosphate buffer (pH 7.0) using Nanosep 300 kDa Omega centrifugal filters (Pall Corp., Ann Arbor, MI, USA). The surface of the NPs were activated with 10 mmol/L sulfo-NHS (Sigma-Aldrich, St. Louis, Mo, USA) and 0.75 mmol/L EDC (Sigma-Aldrich) in 10 mM phosphate buffer (pH 7.0). The antibodies and lectins were coupled to the activated particles in phosphate buffer (10 mM, pH 8.0) by vigorous shaking at RT for 2 h. The bioconjugated NPs were washed with Tris-based buffer (10 mM Tris pH 7.8, 0.05% NaN_3_) and stored overnight at +4 °C with 2 g/L BSA buffer to block the residual active sites on the NPs. The bioconjugated NPs were again washed, resuspended and stored at +4 °C for two days to remove the aggregates. After centrifugation (350 × g, 5 min), the aggregate-free supernatant was transferred in to a new tube and stored at +4 °C.

### Eu^3+^-chelate labeling of the antibodies and lectins

The anti-CD9 and a panel of lectins (Table 1) were labeled with Eu^3+^-chelate as described in the previous publication^31^. In brief, labeling was done by adding 100-fold molar excess of Eu^3+^-chelate over mAb to the reaction mixture containing mAb and 50 mmol/L carbonate buffer (pH 9.8). The reaction mixture was incubated overnight at +4 °C. Unconjugated chelate was removed by gel filtration with a NAP-5, and NAP-10 columns. The labelled antibodies and lectins were stabilized by adding 0.1 g/L BSA and were stored at +4 °C.

### Immunoassay protocols

**Figure 6:**
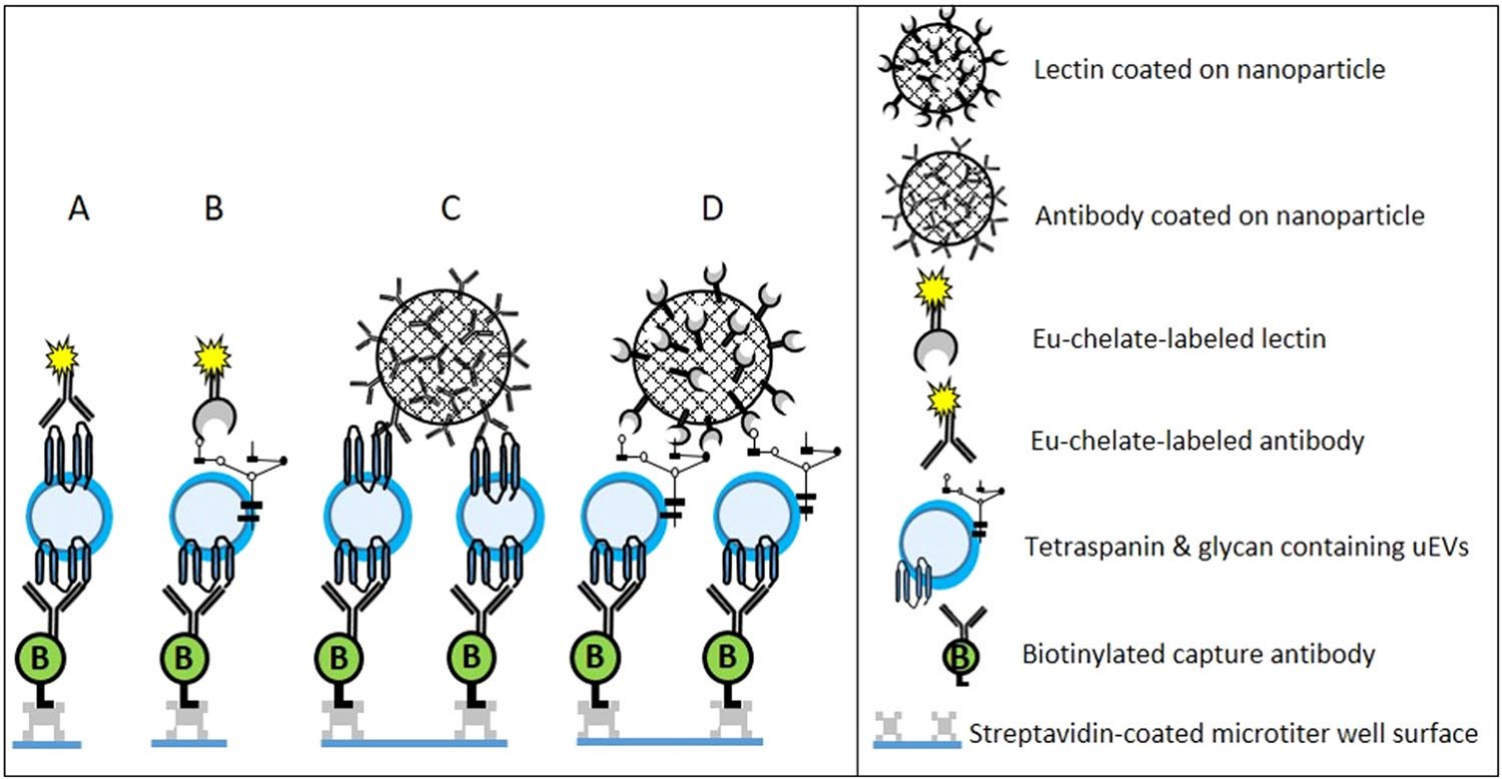
Schematic representation of the established time-resolved fluoroimmunoassay configurations. The biotinylated antibodies are immobilized on the surface of the streptavidin coated microtiter wells for capturing the EVs from the sample. The captured EVs are detected with either Eu^3+^-chelate labeled tracers: (A) antibodies, (B) lectins or Eu^3+^-nanoparticles tracers coated either with (C) antibodies or (D) lectins.

The time-resolved fluorescence immunoassays were performed as shown in the Fig. 6. In brief, biotinylated antibody (200 ng/well) was immobilized in streptavidin-coated wells of a 96-well microtitration plate (KaiSA96, Kaivogen, Turku, Finland) in 25 µL of assay buffer (Kaivogen, Turku, Finland) and incubated for 1 h at RT on a plate shaker at 40 × g. After incubation, the wells were washed 4 times with the wash buffer (Kaivogen, Turku, Finland) and DELFIA plate washer (Perkin-Elmer). The immobilized antibodies were allowed to capture the EVs from 200 µL/well of sample for 1 h at RT on a plate shaker. In case of urine samples, the varying pH of the samples was normalized by adding 20 µL of 10X Trizma in each well along with the 200 µL of urine sample. Conditioned cell culture medium (RPMI with 10% FBS) and EV-stripped urine were used as blanks for the immunoassays involving cell culture supernatant and urine samples, respectively. Then, the wells were washed 4 times with wash buffer in DELFIA plate washer. The detection of the captured EVs was achieved either by 5 × 10^7^/well NPs coated with antibodies or 1 × 10^7^/well nanoparticles coated with lectins in a final volume of 25 µL/well, and incubated for 1 h at RT on a plate shaker. For the Eu^3+^-chelate based assays, 50 ng in a final volume of 50 µL of Eu^3+^ -labeled antibodies or lectins were used with shaking for 1 h at RT. Then, the wells were washed 4 times with wash buffer in DELFIA plate washer. After washing, 200 µL of enhancement solution (Perkin-Elmer, Turku, Finland) was added and incubated again on a plate shaker for 10 min at RT. This was followed by measurement of signals (λ_ex_: 340 nm; λ_em_: 615 nm) using Victor^TM^ 1420 multilabel counter (Perkin-Elmer). All measurements were carried out in triplicate and average of measurement was used in analysis.

## Supplementary information

SUPPLEMENTARY data
