# Supplementary material for: A Nanoparticle-Based Approach for the Detection of Extracellular Vesicles
Source: Sci Rep. 2019 Jul 11;9:10038. doi: 10.1038/s41598-019-46395-2 (PMC6624270; doi:10.1038/s41598-019-46395-2)
Supplement: Supplementary file 1 — SUPPLEMENTARY data [file 41598_2019_46395_MOESM1_ESM.docx]

A Nanoparticle-Based Approach for the Detection of Extracellular Vesicles

**Md. Khirul Islam*^1^, Parvez Syed^1^, Laura Lehtinen^2^, Janne Leivo^1, 3^, Kamlesh Gidwani^1^ Saara Wittfooth^1^, Kim Pettersson^1^ and Urpo Lamminmäki^1^**

^1^Department of Biochemistry, Division of Biotechnology, University of Turku, Turku, Finland.

^2^Department of Pathology, University of Turku and Turku University Hospital, Turku, Finland.

^3^Department of Urology, Erasmus Medical Center, Rotterdam, The Netherlands.

**^*^Corresponding author:** md.k.islam@utu.fi

**Supplementary data**

**
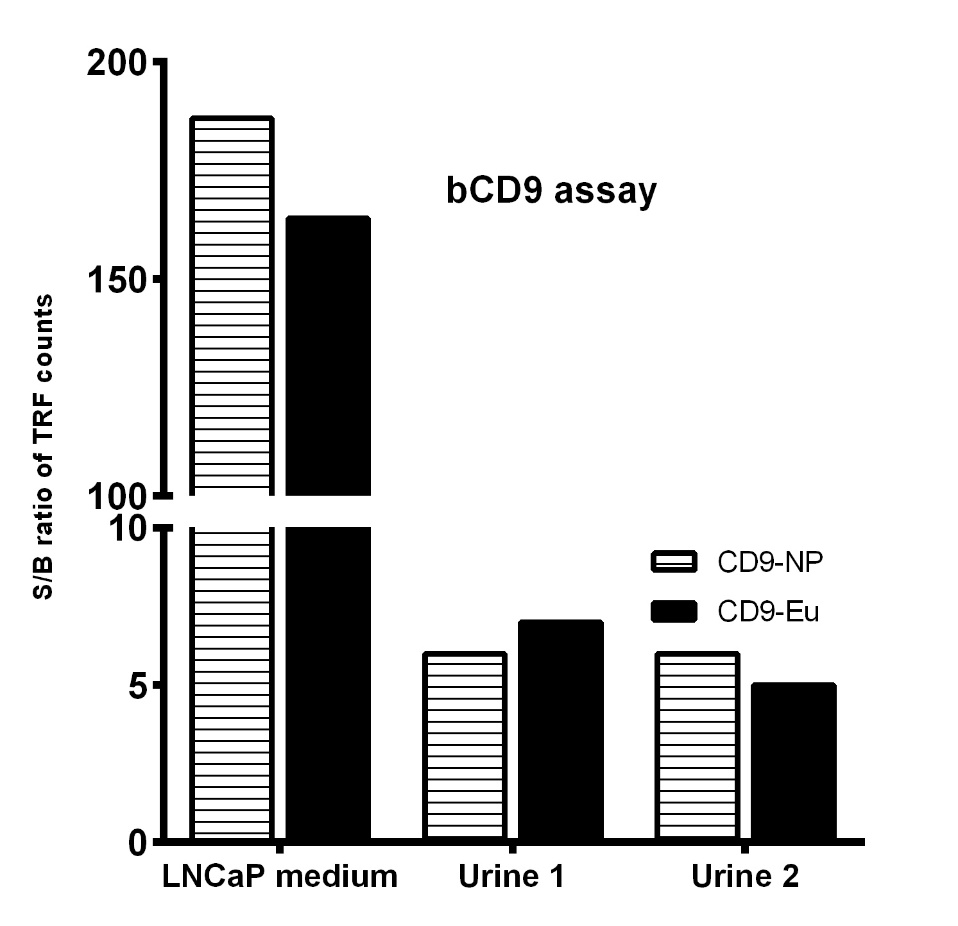
**

**Supplementary Fig. S1:** Comparison of the S/B ratio from CD9-NP and CD9-Eu assays.

## Effect of Tamm-Horsfall protein (THP) on the immunoassay

THP is the most common contaminant found during the process of exosome isolation. Also, the polymerized THP entangles the uEVs by recognizing mannose on the surface of the uEVs. To investigate the effect of THP on the assay, signals obtained from NaCl treated and untreated samples were compared. The S/B ratio obtained from the wells with NaCl treated sample was much lower compared to the untreated sample when Eu^3+^-THP antibody was used. However, no significant differences were observed in the S/B ratios obtained from untreated urine sample and NaCl treated urine sample when detected with CD9-NP, CD81-NP, and CD63-NP (Fig. 6). This experiment showed that the presence of THP in the urine had no effect on the outcome of the assay which indicates that NPs do not interfere with THP.


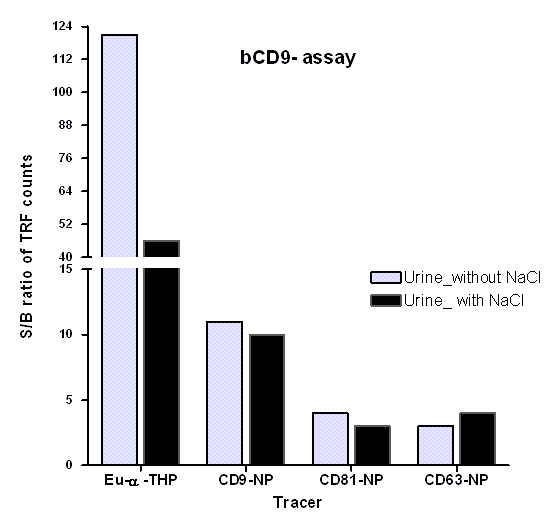


**Supplementary Fig. S2: Effect of THP on the nanoparticle aided TRFIA.** EVs from NaCl treated and untreated urine samples were captured using biotinylated-CD9 antibody. The NaCl treatment indicated considerable loss of THP from the urine samples. However, no signal loss was observed when CD9-NP, CD81-NP, and CD63-NP indicating that the presence of THP in the urine sample has no effect on NPs in the assay.


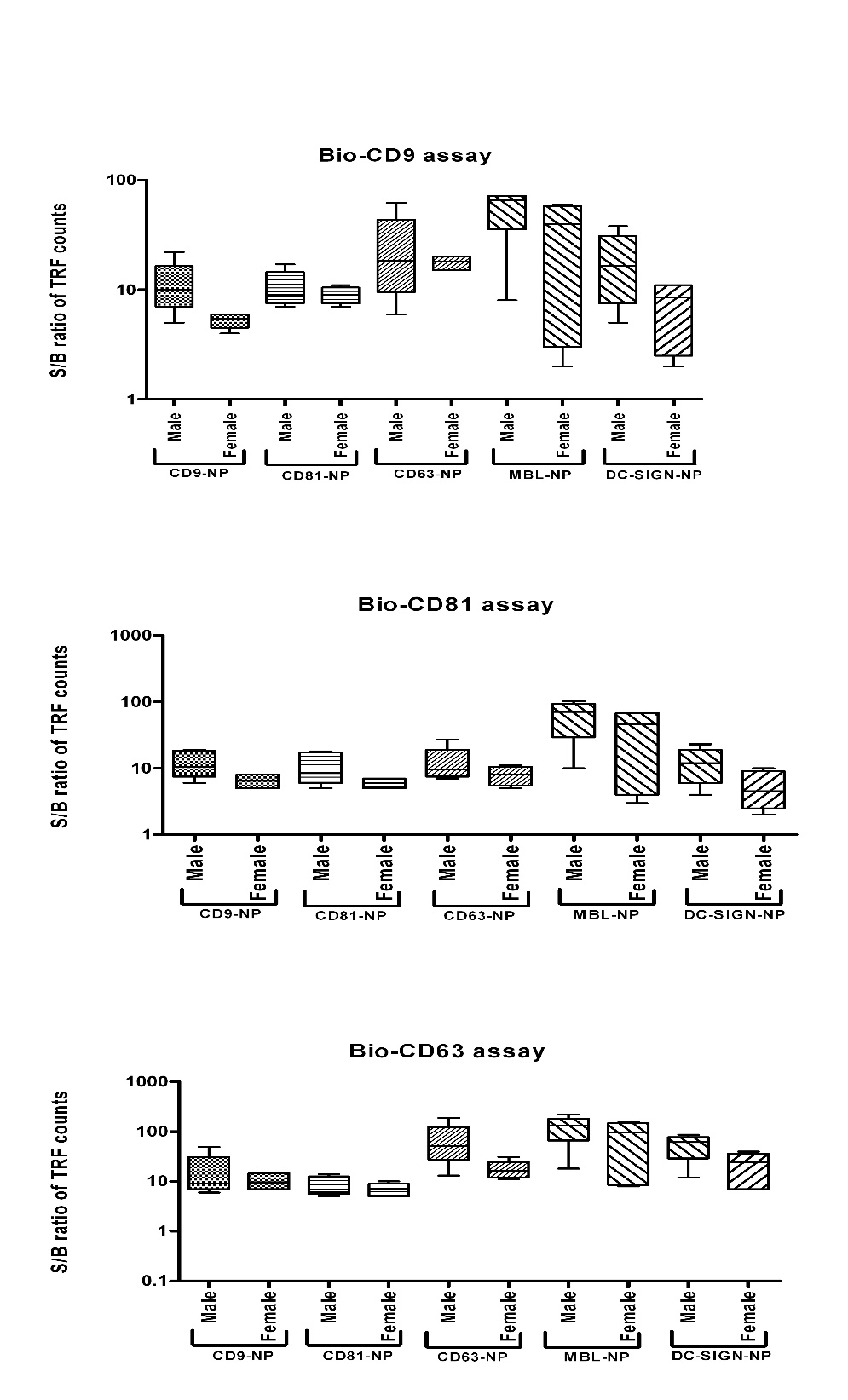


**Supplementary Fig. S3: Comparison signal between male and female:** Signal difference in (a) Bio-CD9, (b) Bio-CD81, (c) Bio-CD63 assays were showed among male (n=6) and female (n=6) urine sample. A total of 200 µL urine was taken for each setting to run the experiment. To get net signal, background signal was subtracted from original signal. All the values were expressed as mean ± SEM.

## Assay reproducibility

In order to check the reproducibility of the optimized assay, immunoassays were conducted on two different days with LNCaP and urine as sources for EVs. For this experiment, biotinylated CD9 antibody was used for capturing the exosomes and the captured EVs were detected using tetraspanin antibody-NPs ans lectins-NP. In both the cases, the signals obtained from two days were almost identical, as shown in the Supplementary Fig. S3:

**R² = 0.9962**

**R² = 0.9982**


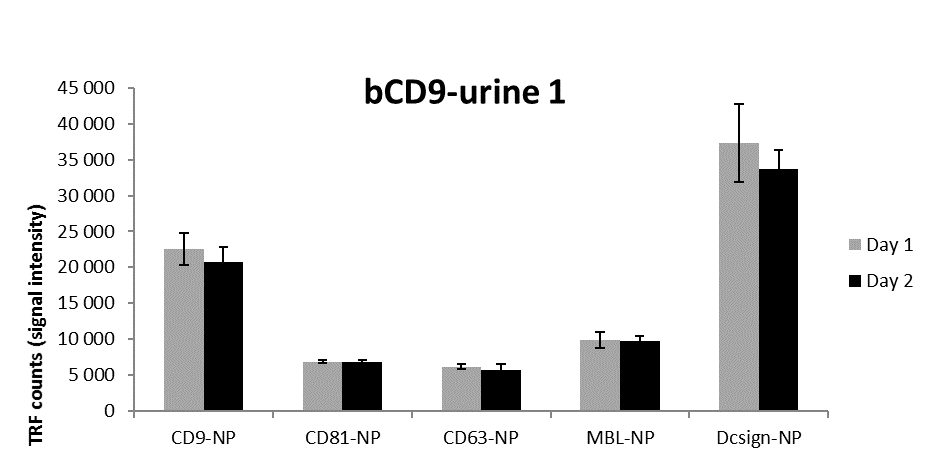

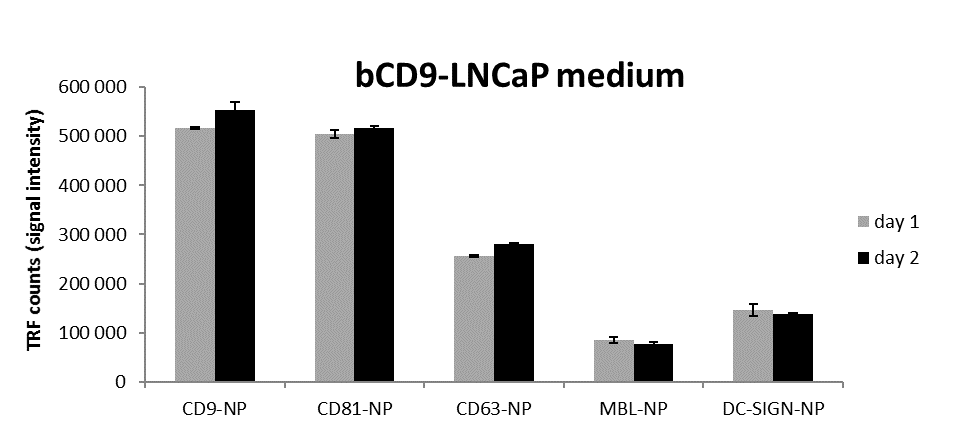


**a**

**b**

**Supplementary Fig. S4:** Nanoparticle-based TRF immunoassay reproducibility. Figure a and b represent the immunoassay results obtained from two different days using LNCaP cell culture supernatant and urine, respectively. Measurements were taken in triplicates and the average counts along with SEM.
